# Comprehensive Analysis of the ILCs and Unconventional T Cells in Virus Infection: Profiling and Dynamics Associated with COVID-19 Disease for a Future Monitoring System and Therapeutic Opportunities

**DOI:** 10.3390/cells11030542

**Published:** 2022-02-04

**Authors:** Elena Lo Presti, Andrea De Gaetano, Giovanni Pioggia, Sebastiano Gangemi

**Affiliations:** 1 National Research Council (CNR)—Institute for Biomedical Research and Innovation (IRIB), 90146 Palermo, Italy; andrea.degaetano@irib.cnr.it; 2 Biomathematics Laboratory, National Research Council (CNR)—Institute for Systems Analysis and Computer Science ‘A. Ruberti’, UCSC, 00168 Rome, Italy; 3 National Research Council (CNR)—Institute for Biomedical Research and Innovation (IRIB), 98164 Messina, Italy; giovanni.pioggia@irib.cnr.it; 4 School and Operative Unit of Allergy and Clinical Immunology, Department of Clinical and Experimental Medicine, University of Messina, 98125 Messina, Italy; sebastiano.gangemi@unime.it

**Keywords:** unconventional T cells, COVID-19, SARS-CoV-2 infection, MAIT, ILC, NKT, gamma-delta T cells, clinical trials advanced therapies, viral respiratory pandemic

## Abstract

This review is a comprehensive analysis of the effects of SARS-CoV-2 infection on Unconventional T cells and innate lymphoid cells (ILCs). COVID-19 affected patients show dysregulation of their adaptive immune systems, but many questions remain unsolved on the behavior of Unconventional cells and ILCs during infection, considering their role in maintaining homeostasis in tissue. Therefore, we highlight the differences that exist among the studies in cohorts of patients who in general were categorized considering symptoms and hospitalization. Moreover, we make a critical analysis of the presence of particular clusters of cells that express activation and exhausted markers for each group in order to bring out potential diagnostic factors unconsidered before now. We also focus our attention on studies that take into consideration recovered patients. Indeed, it could be useful to determine Unconventional T cells’ and ILCs’ frequencies and functions in longitudinal studies because it could represent a way to monitor the immune status of SARS-CoV-2-infected subjects. Possible changes in cell frequencies or activation profiles could be potentially useful as prognostic biomarkers and for future therapy. Currently, there are no efficacious therapies for SARS-CoV-2 infection, but deep studies on involvement of Unconventional T cells and ILCs in the pathogenesis of COVID-19 could be promising for targeted therapies.

## 1. Introduction

The knowledge to discriminate mild and severe disease is critical for fighting the pathophysiology of COVID-19, which is imparted not only by the SARSCoV-2 viral infection, but also by the host immune response that determines acute respiratory distress syndrome in critical cases. 

Several studies identified immune features associated with severe COVID-19 disease, such as lymphopenia [1]; highest production of pro inflammatory cytokines (such as IL-1β, IL-6, and TNF-α) [2]; and early activation and exhaustion of both innate and adaptive immune cells [3]. Conventional T cells are involved in COVID-19 disease, but the role of Unconventional T cells and ILCs remains unclear. Unconventional T cells do not recognize classical peptide antigens (non-MHC-restricted T cells) and they circulate as abundant populations of cells mainly involved in rapid response against pathogens (time of effector function are hours compared to days or week for MHC-restricted T cells) [4]. This group consists of Natural Killer T cells (NKT cells), MR1- restricted mucosal associated invariant T cells (MAIT cells), and γδ T cells, and often they are the majority of T cells in tissues such as the liver and gut mucosa. For this group of cells, we associate the innate lymphoid cells (ILCs), which are not CD3+ and are found in almost every tissue but are specifically enriched at mucosal surfaces with the important role in maintaining epithelial barrier integrity and regulating immune responses. The emerging role of these cells is clear considering that defects and deficiencies in unconventional T cells and ILCs are associated with autoimmunity, chronic inflammation, cancer and infectious disease. Before entering in the description of results on unconventional T cells in severe and moderate COVID-19 patients, we briefly summarize the important features of NKT, MAIT, γδ T cells and ILC involvement in infectious disease [5].

### 1.1. Role of NKT in Virus Infection

Natural killer T (NKT) cells share properties of both T and natural killer (NK) cells [6,7,8]. It is possible to distinguish two main NKT cell subsets both reacting to lipid-based antigens presented by the atypical MHC-I (-like) molecule CD1d on antigen presenting cells (APCs) [9,10]. The best-characterized NKT cell subset is type 1 NKT cells, which express a semi-invariant TCR that combines Valpha24-Jalpha18 in humans. In contrast, type 2 NKT cells exhibit a diverse TCR repertoire. Furthermore, all type 1 NKT cells react to the glycosphingolipid antigen alpha-galactosylceramide (alpha-GalCer) [4,9], while Type 2 do not react to alpha-GalCer and are more present in humans than in mice.

When they exit the thymus, they are precommitted subsets or acquire polarized functions in the periphery [11]. Indeed, NKT cells home to several lymphoid and non-lymphoid organs [12], the mechanisms of which underlying their recruitment to different tissues have not been characterized yet. In humans, there are large inter-individual variations in NKT cell numbers, from 0.01% up to 1%, and rarely up to 5% of the total T cell population in human blood [4].

In HIV infection, high viremia is correlated with lower numbers of circulating NKT cells, though it is possible that NKT cell reduction occurs from the direct or indirect activation by APCs. Recently it was shown that CD1d expression is lower, particularly on CD14+ monocytes, in HIV-infected individuals. That reduction of CD1d is caused by the HIV-1 protein Nef, which physically associates with the cytoplasmic tail of CD1d interfering with its surface expression [13,14] and may negatively influence the ability of NKT cells to recognize infected cells [15].

Recently, a dynamic participation of NKT cells was demonstrated during MCMV (murine cytomegaloviruses) infection displaying signs of activation such as up-regulation of CD25 and up-production of IFN-γ [16]. The hypothesized mechanism of the NKT response was an indirect activation by activated DCs and TLR9 dependent fashion [17]. Moreover, NKT cells have a role in the clearances of the HSV-1 infection. Indeed, though HSV-1 reduces CD1d cell surface expression on APCs and prevents the reappearance of endocytosed CD1d on the cell surface, HSV-1 at low MOI increases CD1d expression on DCs and causes NKT proliferation in vitro. The beneficial role of NKT during the influenza virus was confirmed by a recent publication demonstrating alpha-GalCer as a potent mucosal adjuvant to trigger protection against Influenza A virus (IAV) infection [18]. However, whether endogenous antigens or other ligands drive autoreactive NKT cell responses in infections with viruses is still largely unknown. Indeed, depending on the patho-physiological context, different self-antigens could be presented to NKT cells that affect their functional response [19].

### 1.2. Role of MAIT in Virus Infection

MAIT are defined by co-expression of TCR-Vα7.2 and CD161, and they are predominantly CD4/CD8 double negative or CD8+, and recognize riboflavin metabolites presented on MR1 [20]. There are two subsets of MAIT cells: expressing high levels of CD161 (CD161hi/CD161++) and a population of CD8+ T cells expressing lower, or intermediate, levels of CD161 (CD161int/CD161+) also present in the circulation. Although CD161 is associated with the ability to express IL-17, secretion of this cytokine among CD8+ T cells is restricted to the CD161hi subset. MAIT cells were a major population within the liver, but they infiltrate the gut, especially in patients with inflammatory bowel disease (IBD) [21]. In this case, the MAIT population was enriched for expression of CD103 and together with CD69 indicating as a resident memory T cells. Several ligands presented on MR1 such as 5-(2-oxopropylideneamino)-6-D-ribitylaminouracil (5-OP-RU) and 5-(2-oxoethylideneamino)-6-D-ribitylaminouracil (5-OE-RU) produced by several bacteria, mycobacteria and yeasts during riboflavin (vitamin B2) synthesis can activate MAIT and represent effective host-pathogen defense. Moreover, MAIT cells have a constitutive effector-memory phenotype, (CD45RA-CD45RO+ CD95HiCD62LLoCD44Hi), due to secretion of a wide variety pro-inflammatory cytokines and they populate human tissues, typically comprising 1–4% of all T cells in peripheral blood, up to 10% of airway T cells, and 20–40% of liver T cells [22]. MAIT cells can also be activated by cytokines, especially IL-7, IL-12, IL-15, IL-18, and type-I IFNs, acquiring the ability to respond to viruses by production of IFN-γ. Indeed, patients hospitalized with the H7N9 strain of influenza A virus showed higher MAIT cell frequencies and those correlated with subsequent recovery. It has been observed that cytokine-activated MAIT cells could reduce replication of hepatitis C in vitro in an IFN-γ dependent manner. However, the MAIT defense against virus infection was dependent on TCR more than cytokines as confirmed in immunodeficiency-infected mice [23].

### 1.3. Role of γδ T Cells in Virus Infection

A small proportion (1–5%) of circulating CD3(+) T-lymphocytes consists of T-lymphocytes expressing the γδ T-cell receptor (TCR). Circulating γδ T lymphocytes express prevalently Vγ9Vδ2-encoded TCR, and Vδ1 T lymphocytes are resident in the skin, lung, intestine, and colon epithelia [24], while expression of Vδ3 gene can be associated with Cytomegalovirus (CMV) infection or B cell leukemia. In the absence of processing, presentation and major histocompatibility complex (MHC) restriction, human Vγ9Vδ2 T cells recognize phosphoantigens (PAgs), intermediates of the mevalonate pathway [25,26]. Concentrations of PAgs necessary for activation of Vγ9Vδ2 T cells are achieved after infection or tumor transformation and not under physiological conditions [27]. Therefore, Vγ9Vδ2 TCR acts in a similar manner to a pattern-recognition receptor that detects metabolic changes observed in transformed or infected cells.

Moreover, by means of VΦΝ2-specific mAb, it has been possible to detect another subset of γδ T called Vγ9Vδ2+ T cells clonally expand in response to CMV but the range of pathogens to which they respond is still unclear. Based on Davey’s work [28], Vγ9Vδ2+ T cells responses could contribute when conventional immune subsets are suppressed in several clinical scenarios. The γδ T cells participate in the first line of immune defense against virus infection. For example, in HIV infection, in which γδ T cells are the major T cells circulating, the partial switch of Vδ2 T cells repertoire towards Vδ1-expressing cells is probably caused by depletion of Vδ2-expressing cells [29]. Recently, the involvement of Vγ9Vδ2 T cells in influenza disease due to their presence in lung tissues [30] was demonstrated. Indeed, the γδ TCR repertoire found upon influenza virus- stimulated was Vγ9Vδ2 TCRs (80%) and these cells produced IFN-γ.

The cytotoxic activity of infiltrating γδ T cells in the lungs may even be significant in the early stages of M. tuberculosis infection by limiting the amount of bacilli and favoring the development of the protective γδ T cells immune response. The γδ T cells provide a critical early burst of IFNγ that conditions dendritic cells (DC) for efficient priming of CD8 T cells and for the full development of a protective response [31]. This protective role has been demonstrated also in CMV infection and Herpesvirus infection [32].

### 1.4. Role of ILC in Virus Infection

ILCs were divided into three groups: group 1 ILC (ILC1 and NK cells), group 2 ILC (ILC2s), and group 3 ILC (ILC3s and LTi). They are distinguished based on their cytokine production patterns that correspond to the helper T cell subsets Th1, Th2, and Th17, respectively. Despite the fact that this classification has been generally accepted by researchers in this field, ILC subsets continue to be extensively studied. ILC3 cells could mediate colitis in mice lacking T cells and [33] and as opposed, ILC2 cells predominate in the lung [34], though it has been increasingly recognized that ILC3s play a role in lung immunity. ILC populations require IL-7R signaling and derive from Id2 expressing progenitor cells [5]. It is possible to distinguish ILC population by transcription factor as Tbet for ILC1, GATA3 for ILC2 and RoRγt for ILC3s. Moreover, ILC1 subset lacks expression of ckit (also known as CD117) and produces prevalently IFN-γ and TNF-α, while ILC2 produces type-2 cytokines (IL-5, IL-9, IL-13) in response to extracellular parasite infections. Although ILCs play roles in several autoimmune diseases, they are involved in clearance of infected cells. The CXCL13-CXCR5 axis has been implicated in localization of ILC3 to inducible bronchial-associated lymphoid tissue (iBALT) developing during M. tuberculosis infection in mice [35], while CXCR5 and CCR6 were expressed by ILC3s recruited to sites of lung tumors in patients [36]. In the gut, IL-1β and IL-23 stimulate ILC3s to produce IL-17 and IL-22 [37], which in turn regulate epithelial barrier function and mediate host response to infections, and in the lung they can rapidly produce the same cytokines upon stimulation of bacterial pneumonia or viral lung infections [38].

## 2. Search Strategy

Our search strategy was based on looking for single key terms together with the term “COVID-19” in the title and abstract of a reference using the NCBI (PubMed) database. Data collection was performed by searching the following key words: “COVID-19” AND “NKT”; “COVID-19” AND “MAIT”; “COVID-19” AND “gamma-delta T cells”; “COVID-19” AND “ILC”; “COVID-19” AND “Unconventional T cells”. Even though many papers discuss COVID-19 considering several facets, we excluded those that did not directly analyze these subpopulations in different SARS-CoV2 infection stages and the studies on mouse models or other animals. We found 9 papers for NKT; 11 papers for MAIT; 5 papers for γδ T cells (one is not in the table) and 4 papers for ILCs. Using different combined keywords, some papers were counted more times but were included in our count for class of cells because of the papers’ discussion about Unconventional T cells and were included in the search of “COVID-19” and “Unconventional T cells”. Papers were collected in October 2021.

## 3. Results

Based on observations obtained from literature, we next explored retrospective studies on COVID-19 involving patients with several types of symptoms: mild, moderate, severe and critical, and we considered also the papers discussing recovered patients. For deep learning about COVID-19 infection, we detailed which type of control authors used: healthy subjects or ill subjects in ICU or non-COVID-19 but hospitalized. Special consideration was given for the type of sample involved relating the results to the age and gender. Finally, we highlighted methods used to perform experiments.

### 3.1. NKT

We collected 9 papers about NKT in COVID-19 patients with severe (9/9 papers) and non-severe (8/9 papers) symptoms. Only 4/9 papers evaluated recovery patients and only 2/9 used as control non-COVID-19 patients together with Healthy subjects (HCs). All papers except one had a cohort homogeneous with more males than females; a part one paper had a cohort composed of pregnant women (Table 1). The analyses were performed on blood by Flow cytometry (5/9 papers) and Sc-RNA-seq (4/9 papers) and on serum analyzed by Luminex or ELISA.

Patients admitted to the ICU for severe COVID-19 had a reduced circulating NKT percentage compared to non-COVID-19 patients and HCs [39] and compared to patients with moderate symptoms [40], even if the latter data is not significant. Absolute number of NKT decreased significantly compared to HCs [41,42], while for Parrot the absolute count of NKT was largely unchanged [40]. In moderate patients, NKT percentage was slightly high compared to severe patients and HCs, while the absolute count was unchanged [40,43] and in other papers the absolute number of NKT increased significantly compared to severe patients and HCs [41,42]. 

**Figure 1:**
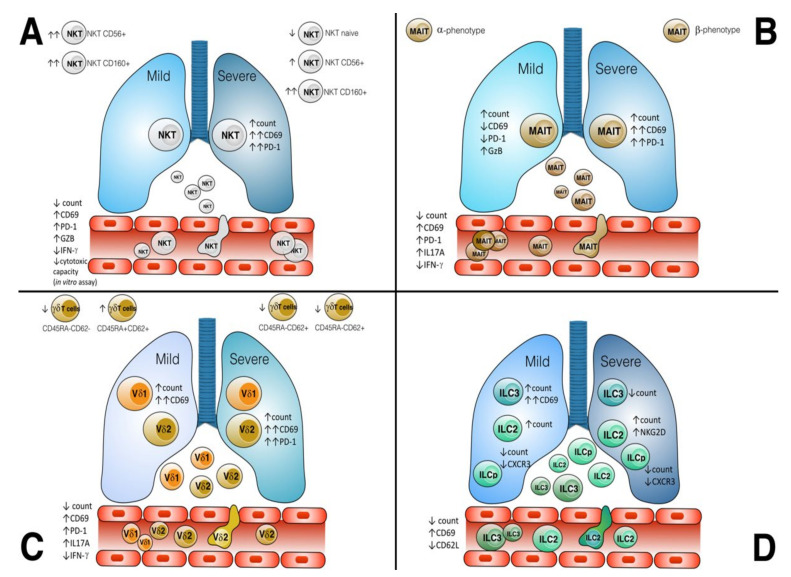
Phenotypic and functional alterations in Unconventional T Cells and ILC in SARS-CoV-2 infection. (**A**) The host response of NKT cells to SARS-CoV-2 infection is illustrated with modulation of surface receptors distinguished in circulating or recruited in lung tissue as well as the phenotypic subsets divided for symptoms in moderate and severe subgroups. (**B**) Specific features of MAIT cells associated with moderate and severe COVID-19 and the phenotypic subsets revealed. (**C**) The host response of γδ T cells to SARS-CoV-2 infection is illustrated with modulation of surface receptors in circulating and infiltrating γδ T cells as well as the phenotypic subsets divided for symptoms in moderate and severe subgroups of γδ T cells subsets (Vδ1 and Vδ2 T cells). Moreover, phenotypic subsets of γδ T cells were evaluated in severe patients. (**D**) ILC precursor (ILCp), ILC2 and ILC3 responses were illustrated with expression markers of moderate and severe patients and from ILC in blood and in lung tissue.

Regarding the activation state, CD69+ expression on NKT was higher in COVID-19 patients compared to HCs and non-COVID-19. NKT CD69+ correlated positively with plasmatic IL-18 and with decreasing hypoxemia [39] (IL-18 plays an important role in the induction of IFNγ production by T cells and NK cells). Importantly, discharged patients showed an increased level of NKT CD69+ compared to non-discharged at 15 days [39]. However, Zhang et al. evaluated the presence of three different subgroups of NKT with activated phenotype: NKT CD56+ and NKT CD160+ that increased in the same type of samples compared to HCs and exhausted markers in NKT CD160+ increased in Severe patients (See Figure 1A). In moderate patients, there were all three subgroups up-represented, while the naïve group (CCR7+SELL+) decreased in severe COVID-19 patients. Surprisingly, CD56+ and CD160+ NKT subgroups remained in recovery patients.

The cytokine production was evaluated upon in vitro stimulation [39,44], while cytotoxic activity was evaluated by cells’ coculture (effector:target cells) [45]. Data collected revealed that circulating NKT from COVID-19 patients produced significantly less IFN-γ but more IL-17 compared with cells from healthy donors even if these cytokines were more represented in ETAs than plasma [39], and decreased the production of IL-12A and IL-10 compared to control [44] (See Table S1). CD3+CD56+CD16+ cells expressed CD107a,b but reduced their ability to synthesize GZB in response to Hsp70 peptide. The CD56+NKT subset expressed GZMA as well as CD160 subset but the latter showed higher exhaustion scores than those of the other subsets prevalently in severe patients [46]. Moreover, the specific expression of FCGR3A in NKT CD160+ indicated an antibody-dependent cell-mediated cytotoxicity activity [46] as well as NKT CD56+, and CD160+ showed migration capability compared to NKT naïve [46]. From ETA samples, NKT cells expressed CXCL10 and CXCL12 [39], while CXCR5 and CXCR3 showed the mean of expression of only 0.75-fold more.

Finally, a particular aspect was treated by Chen et al. [47] that enrolled 179 pregnant and nonpregnant women COVID-19 patients. The Gene expression analysis showed a T cell activation and low type I interferon production in severe patients, while in Moderate patients there was a response to the virus by IFN-γ production. Pregnant patients had more activated NKT compared to nonpregnant patients, and it was associated with leukocyte cell-cell adhesion genes enriched.

### 3.2. MAIT

We collected 11 papers about MAIT in COVID-19 patients with severe (11/11 papers) and non-severe (10/11 papers) symptoms. Only 9/11 papers evaluated recovery patients, and only 3/11 used as control non-COVID-19 patients together with healthy subjects. All papers except one had a cohort homogeneous with major males than females—a part one paper, in which the cohort was composed of pregnant women. In one paper, gender was not specified (Table 2). Complex analyses were performed on blood by Flow cytometry (7/11 papers) and Sc-RNA-seq (8/11 papers) and on serum analyzed by Luminex or ELISA.

Flow cytometry analysis showed that reduction of MAIT cells was significant in severe COVID-19 patients compared to healthy controls [39,40,43,46,48,49,50,51,52,53] and compared to HCs or infected patients. In convalescent patients, MAIT cell frequency did not significantly increase. COVID-19 patients with mild symptoms showed a lower percentage [43,51,52] or a slightly higher one [48], or much more than in severe [40,46,49,53]. This data correlated with the low percentage of CCR6+ CCR7+ MAIT, suggesting a migration into inflamed tissues and/or activation-induced cell death. Sc-RNA-seq analysis revealed a depletion of MAIT cells in patients with severe disease even if the number of clonotypes of MAIT cells was relatively high. Moreover, there were more large clonal expansions (clonal size > 10) in the severe cases than in the other conditions [50].

MAIT cells showed higher expression of CTLA4 and PD1 in severe COVID-19 but not significantly [43], and in convalescent patients with severe and mild symptoms CTLA expression decreased deeply [43]. CD69 and PD1-expressing MAIT were significantly higher in ETA compared to the blood of the same patients [39]. In the same way, CD38, CD69 and HLA-DR were expressed in MAIT from severe patients more than healthy controls [40,43,52] without any correlation with sex or phenotype [51]. 

In particular, CD69 was slightly lower in mild patients in percentage and MFI [48], while CD69 on MAIT cells of dead patients was higher than in patients who survived [40]. Interestingly, CD69 expression on MAIT positively correlated with the level of plasmatic IL-18 that was significantly higher in the plasma of long-term ICU patients with fatal COVID-19 [39,53] compared to plasma from patients hospitalized in an ICU or IDU [53]. Considering the group of pregnant COVID-19 patients [47], data showed significantly reduced frequency of MAIT compared to healthy pregnant patients and did not increase in convalescent pregnant COVID-19 patients. 

Regarding the cytokine production, Notarbartolo et al. found that TC17/MAIT cells were exclusively expanded in patients with mild symptoms, both during the infection and post-infection [49]. A deeper analysis based on in vitro assay upon stimulation with *E.coli* [43,52] showed high production of IFN-γ without an upregulation of IL-17A and TNF-α expression [43] as if activated MAIT cells were functionally impaired [52]. 

When MAIT cells were stimulated with IL-12/IL-18 [48,52], there was a decrease in IL-17A and TNF-α compared to healthy controls and an increase in IFN-γ production [43,53], even though this finding was not the same for Hubrack et al. [48]. Upon in vitro stimulation with Iono/PMA, MAIT from mild patients lowered IFN-γ production and increased GzB compared to HCs [53], while MAIT from severe patients increased IFN-γ, GzB and IL-17 (Figure 1B). MAIT from deceased patients produced more IFN-γ compared to surviving patients [53]. DEG analysis of genes related to cytokines expression of MAIT revealed that from patients in ICU there was a reduction of IFNA and IL18 in opposite to IDU [53]. However, another transcriptional profile indicated that MAIT cells were the main subset of airway T cells expressing IL17A [40]. This profile was paired with an expression of TNF and an apparent lack of IFNG and GZMB transcripts [40]. Interestingly, in asymptomatic carriers with COVID-19 the function of MAIT remain unchanged and upon *E.coli* stimulation or IL-12/IL-18, convalescent patients restored their functions [52].

### 3.3. γδ T Cells

We collected 4 papers that analyzed γδ T cells frequency and functions in patients with COVID-19 with different symptoms: severe (4/4 papers) and non-severe (3/4 papers). Data from non-COVID-19 at ICU (2/4 papers), healthy control (4/4 papers), and recovery patients (3/4 papers) were used as controls (Table 3).

Studies on γδ T cells during COVID-19 infection are an attractive topic, given the opportunity to easily expand and manipulate γδT cells in vitro. We observed that the authors do not always consider both subsets of γδ T cells that differentially can contribute to defense against virus infection and be used for possible immunotherapies. 

Severe patients with noninvasive or invasive ventilation were evaluated to reduce the absolute numbers and percentage of circulating Vγδ+Vδ9+ compared to MD and HCs [41,44,46], though there was a slight decrease for the Vδ2 subset, which ordinarily dominates blood γδ T cells, a moderate increase for the Vδ1Vδ2- subset and no change for the Vδ1 T cells subsets evaluated by Jouan et al. [39]. The latter data suggest substantially shifted γδ T cells composition toward Vδ1+ cells subsets [54] (not in table). On the other hand, mild patients showed that the absolute number of γδ T cells increased little compared to severe patients, though not significantly, and they did not increase compared to HCs [41,44,46]. Recovered patients did not demonstrate any significant alterations in frequencies of the different subtypes of γδ T cells in relation to disease clearance [41], but only Vδ1Vδ2- T cell subsets were lower in patients still in the ICU at 15 days compared to discharged at 15 days [39].

Activation state and functions (See Table S2) were evaluated considering the expression of CD69, CD45RA and CD62L markers in circulating [39,41,44,46] and infiltrating γδ T cells [39,41] that at low levels expressed chemokine receptors such as CXCR5, CXCR3 [44] and CXCL10 and CXCL12, respectively, in blood and ETA. Regarding the activation state, levels of CD69 and PD-1 expressing γδ T cells were significantly higher in ETA compared with the blood of the same patients [39], while any significant change was for PD1 expressing γδ T cells subsets in blood [39]. CD69 and PD1 expressing Vδ2+ T cells showed a higher level in severe patients compared to HDs, while CD69 expressing Vδ1 T cells but not PD1+ Vδ1 showed no significant change in severe patients compared with non-COVID-19 critically ill controls. The Vδ1Vδ2 did not change compared with non-COVID-19 critically ill controls or HDs [39] (Figure 1C). 

Adding to these evaluations, it has been observed that both frequencies and absolute cell numbers of naïve-like γδ (γδ naïve) in COVID-19 patients increased while the other subsets decreased in frequency and absolute numbers [41]. Interestingly, an analysis of the cell cycle showed that frequencies of γδ T cells (mostly Vδ1+ subset) in G1 increased, although few transitioned into S-G2/M [54]. Finally, circulating γδ T cells decreased significantly in IFN-γ production and increased IL-17 compared to HDs [39], while gene expression of circulating γδ T cells between healthy donors and individuals with COVID-19 showed the IL1B was expressed as well as IL1A and TNF [44], though the levels of IL-1β, IL-6, IL-1RA, IFN-γ and IL-17 in supernatants of endotracheal aspirates (ETAs) were higher than serum [39].

### 3.4. ILC

We gathered 4 papers discussing the involvement of ILC and their subsets (ILC1, ILC2, ILCp [55,56,57,58] and ILC3 [59] in COVID-19 patients with severe or moderate symptoms. Only one study considered as control non-COVID-19 patients and evaluated recovered patients; the other ones used as controls only the Healthy subjects (Table 4).

The absolute number and frequency of total ILC decreased in the blood of COVID-19 patients compared to HCs [55,56,57]. A deep analysis on the subsets demonstrated that the absolute number of ILC1 (CD117-CXCR3+) and ILCp (CD117-CRTH2-) decreased in COVID-19 patients as well as ILC2 only in severe patients compared to moderate [55]. Gomez et al. demonstrated a significantly increased frequency of ILC2 [57], while ILC1 and ILC2 classified as CD117-increased on the total of ILC in moderate patients compared with HCs [57]. ILCp decreased in COVID-19 patients but without significance, while the absolute number of ILC type-3 (Lin CD127+CD117+CD294-) increased compared to Severe patients [59]. However, individuals recovering had a 2.39-fold increase in ILC abundance as compared to time of acute disease [56] and had an expansion of CCR10 expressing ILC2 [58] as well.

A specific set of markers were evaluated in ILC (See Table S3); in fact, in COVID-19 patients CD56 was decreased in ILC1, while in ILC2 and ILCp was increased CD69 and only ILC2 decreased CD62L [55] (See Figure 1D). In addition, ILC2 increased NKG2D in severe patients to compared mild and control patients, while any differences in NKG2D expression were observed in ILC1s or ILCPs. The percentage of activated (CD69+) total ILCs and activated ILCp positively correlated with serum IL-6 levels and with CXCL10 levels in the COVID-19 patients. In contrast, there was a negative correlation between CXCL10 and CXCL11 levels and the percentage of CXCR3+ ILCs in COVID-19 patients. Interestingly, ILCs from the pediatric COVID-19 cohort decreased with age, but no one difference in abundance of the ILC subsets was associated with hospitalization [56].

## 4. Remarkable Considerations and Conclusions

Our analysis tried to outline the differences or the equivalences into data collection among several Unconventional T cells and inside each subgroup. 

In ICU hospitalized severe patients, overall evaluation shows that the absolute number and frequency of Unconventional T cells and ILCs decreases compared to the level found in moderate patients and healthy controls but not in all studies were data significant. Frequency was detected unchanged in a few studies [43,52], probably influenced by the number of samples or by intrinsic diversity into the cohort (age, sex, time of sampling). A different type of evaluation using gene expression analysis demonstrated higher NKT levels in severe and critical patients (requiring mechanical ventilation) supposing that a more deepened but expensive analysis could detect new unconsidered aspects [44] (Table S1). 

Data collected by analysis on Unconventional and ILCs from moderate patients were nonhomogeneous. Major studies demonstrated that NKT, γδ T cells and ILCs slightly enhanced their level compared to severe patients, though data were not always significant. These data permit an association with the presence of ILC and Unconventional T cells to moderated symptoms, highlighting their involvement during the infection. All the analyses focused on activation and exhausted markers were helpful in explaining the discrepancy data among severe and moderate COVID-19 patients. Indeed, all circulating cell types exhibited a heightened activation and exhausted state (high expression of CD69 and PD1) that, in the end, directed them towards apoptosis death (Table S2). Another common feature of ILCs and Unconventional cells is cytokine production skewed toward IL-17a instead of IFN-γ with increased production of GZB in severe patients. The cytokine production such as IFN-γ, TNF-β, IL-17A, and GRZ-B did not differ between Unconventional T cells isolated from patients with mild or severe COVID-19 [43].

Another explanation for their decreased blood level is their recruitment in inflamed lungs. In brief, while circulating Unconventional T cells and ILCs were easily analyzed by flow cytometry or gene expression, very few studies evaluated whether those cells infiltrated tissue. In general, there is a marked increase in the levels of MAIT and γδ T cells and ILCs (especially ILC2) in COVID-19 endotracheal aspirates [39,56]. So controversial is the presence in the airway NKT that in some studies they are virtually undetectable [39], while others are detectable as CD56+ and CD160+ subsets [46]. However, CXCR3 remained the marker always evaluated because it is a chemokine receptor that is highly expressed on effector T cells and plays an essential role in T cell trafficking and function. There is a reduction in the percentage of ILCs [55], MAIT [53], NKT expressing CXCR3+ (and an increase in CCR4+), but it is expressed in γδ T cells [44] in both moderate and severe COVID-19 patients (Figure 1). Among the three interferon-inducible ligands, CXCL9 (MIG), CXCL10 (IP-10), and CXCL11 (I-TAC) were evaluated. CXCL10 and CXCL11 levels were higher in airway samples than in serum [39], and they correlated negatively with the percentage of Unconventional T cells expressing CXCR3 [55].

In contrast, CD69 expression increased more in infiltrating Unconventional T cells [39,48]. Indeed, the correlation between CD69 expression of MAIT cells and CXCR3 expression was frequently evaluated [40,53]. Moreover, CD69 expression was related to severe symptoms [39,43,46] and reported higher levels on MAIT cells in deceased patients [40], and ILC and MAIT did not correlate with IFN-γ production.

Thus, SARS-CoV-2 impaired the effector function of Unconventional T cells, particularly in IFN- γ response, and this aspect reflected more severe conditions than moderate. Moreover, the IL-17 production by ILC and the other three groups of cells could suggest a deleterious pro-inflammatory role in severe disease (Table S3). Interestingly, MAIT cells increased IFN- γ expression upon in vitro *E.coli* stimulation significantly and failed to upregulate expression of IL-17A and TNFα, but it was the opposite under IL-12/IL-18 stimulation. Considering that serum and ETA samples of COVID-19 patients were found to have increased levels of IL18, the results obtained by these in vitro experiments highlight crucial therapy applications. Indeed, epigenetic changes observed in patients infected with SARS-CoV-2, such as levels of citrullinated histone H3 (Cit-H3), were elevated [60] and positively correlate with increased cytokines, leukocyte, granulocyte, and platelet counts in COVID-19 (Table S4). Along similar lines, this aspect in Unconventional T cells could be explored to discover promising targets for potential therapeutic strategies [61]. Indeed, histone modifications are implicated in the regulation of IFNs, TNFs, and interferon-stimulated genes (ISGs), and hence innate immune response.

An unexplored aspect regards the role of miRNA in modulating Unconventional and ILC response of severe patients compared to mild patients. This issue is quite studied in cancer with promising results in the association with microbiota in the tumor microenvironment [62]. Indeed, a study by Arisan et al. [63] revealed that miR-8066 elevates the cytokines of PRLR, CXCL6, IL-6, and IL-17 during the infection, while many other ones can mitigate the pathogenesis of COVID-19 disease via binding to the SARS-CoV-2 genome and inhibiting its post-transcriptional expression [64].

The study on recovery patients is an attractive topic that we want to highlight, given the possible consequences of the virus infection on the patients. Indeed, only for MAIT, NKT, and ILC (particularly ILC2 subtype [58]), individuals recovering from COVID-19 restored frequency and absolute number as compared to the time of acute disease. Additionally, discharged patients show higher levels of CD69 compared to those who remained in critical care. Further stratification of COVID-19 patients based on moderate and severe symptoms revealed that MAIT cells from recovered moderate patients looked like they did not restore their frequency. Surprisingly, not all Unconventional T cells, such as MAIT cells, recovered their ability to produce IFN-γ and the expression of CXCR3 after infection though maintained CD69 and PD1 expressed.

It means that SARS-CoV-2 infection affects the response of Unconventional T cells at several levels even after the disease clearance. It was an example of the fact that MAIT cells respond in a different way to different stimulation [43,48] in a different cohort of samples. Today we do not know if, after some time, the response was restored, and thus we auspicate that this type of study will be done as soon as possible.

Importantly, new aspects are emerging about COVID-19 pathogenesis in the innate immune response [65]. However, they remain unanswered: how the virus activates Unconventional T cells or ILCs since it does not directly encode antigens such as Vitamin B metabolites, lipid antigens, or phosphoantigens. Instead, the activation is probably TCR-independent; in fact, TCR levels do not change [39] or respond to a bacterial antigen in case of comorbidity in severe COVID-19 infection. Indeed, it is not clear if the comorbidities can influence the immunological dynamic of the COVID-19 infection since, among severe and moderate patients, there are significant differences in comorbidities. For example, the main comorbidity detected in severe COVID-19 patients was hypertension and the second pathology revealed was diabetes. However, microbial coinfection more than fungi coinfection affected a not too small group of severe patients and was not detected in mild patients [52]. Therefore, the latter difference could be investigated with the viral load that could affect specific responses. COVID-19 development does not happen due to a single molecule but through a heterogeneous immunological response made by different types of cells involved; hence, many studies tried to carry out more categorized analyses about various marker expressions showing a huge diversification of Unconventional T cells in subgroups (See tables).

Based on these considerations, several promising therapies may be beneficial for COVID-19 treatment and can provide research perspectives for SARS-CoV-2 infection [1]. IL-7 immunotherapy is currently being evaluated as a treatment to reverse the lymphopenia in COVID-19 patients, with good results for critically ill COVID-19 patients [66]. Hubrack et al. [48] analyzed the in vitro effect of IL-7 on MAIT cells of only mildly affected patients, testing the possibility of recovering the MAIT cells’ function during earlier stages before the development of further complications. As discussed by Monneret et al. [67], this therapy could enhance the functionality of MAIT cells, increasing perforin levels without significantly enhancing TNF-α and IFN-γ production. A clinical trial in phase II exists to evaluate the safety and tolerability of ex vivo expanded gamma delta T cells (TCB008) in patients with COVID-19 (ClinicalTrials.gov Identifier: NCT04834128).

Another strategy was suggested by Brufsky end Lotze in which ZA or other amino bisphosphonates could be used to immunostimulate γδ T cells. ZA treated dendritic cells could enhance NK activation and expansion as well as prevent expulsion of lysosomes containing SARS-CoV-2 virions [68]. Furthermore, efficient therapies for Hypercytokinemia such as IL1 and IL6 receptor antagonist (Tocilizumab) or p38 and MAPK inhibitors are currently used. But other therapies have been hypothesized such as histone deacetylase therapy or blockading PD1 or PDL1 to prevent lymphocytes exhaustion [69] or through the immunomodulation of other immunological checkpoints (TIM3, CTLA4, LAG3) that now are under study [70]. Indeed, the final aims of the main therapeutic strategies are: to inhibit lymphopenia and compensate the lymphocyte counts in severe patients of COVID-19 and enhance the functionality of cytotoxic cells avoiding cytokine storm effects. In conclusion, although many valuable reviews on Unconventional T cells or ILCs are published [71,72,73], our review summarized and discussed the recovered data connecting them to each other and highlighted that Unconventional T cells and ILCs possess potent effector and regulatory functions. More understanding about the protective or pathogenic roles of various immune cell types and their relationships is needed to improve therapeutic approaches.

## Figures and Tables

**Table 1:** Characteristics of cohort of COVID-19 patients in studies on Natural Killer T (NKT) cells.

	Adult/Child	Severe	Non-Severe	Non-COVID-19 in ICU	Recovered	Healthy Subject
**Jouan 2020 JEM [39]**	Adult. *N* = 30 pts in ICU for severe COVID-19	*N* = 30 pts in ICU 66.7% with IMV	ND	*N* = 17 critically ill pts without pneumonia, requiring IMV.	*N* = 14 pts	*N* = 20 subjects
	↑males↓females	MA: 64 y		MA: 64 y		age- sex-matched
	MDS: 10 d	75% males		55% males		
**Parrot 2021 Science Immunology [40]**	Adult. *N* = 69 pts with AD or C. from Atlas cohort or Biobank.	*N* = 15 from Atlas blood pts + N = 14 from Biobank.	*N* = 9 samples from Atlas blood pts	ND	*N* = 45 pts *N* = 23 convalescent pts from mild disease. *N* = 22 convalescent pts from moderate/severe	*N* = 14 subjects SARS- CoV-2 IgG seronegative
	↑males↓females	MA: 57 y for both cohorts	MA: 56 y			
	MDH: 17 d for Atlas and 34 d for Biobank;	80% males for Atlas samples	67% males		48% males in Mild 82% males in Mod/Sev/conv	
**Odak 2020 eBioMedicine [41]**	Adult. *N* = 30 hospitalized COVID-19 pts.	*N* = 15 pts with non-IMV	*N* = 15 pts with stable parameters with no oxygen flow	ND	*N* = 7 pts Sampling: weeks after the resolution of infection	*N* = 60 matched Healthy Controls
	↑ males↓females	MA: 60 y	MA: 68 y			MA: 54 y
	Mean of days after onset of symptoms: 11 d	86% males	73% males			80% males
**Tomi 2021 Frontiers of immunology [42]**	Adult. *N* = 41 pts with moderate and severe symptoms.	*N* = 20 pts hospedalized and 60% needed IMV.	*N* = 21 pts with mild symptoms without IMV	ND	ND	*N* = 16 healthy volunteers
	↑ males↓females	MA: 62.5 y	MA: 55 y			
		60% males	57% males			
**Deschler 2021 Viruses [43]**	Adult. *N* = 43 hospitalized COVID-19 pts. ↑ males↓females	*N* = 21 pts in ICU 19/21 pts IMV MA: 65 y 76.2% males	*N* = 22 pts MA: 62 y 54.5% males	ND	ND	*N* = 25 MA: 28 y 52.0% males
**Stephenson 2021 Nature medicine [44]**	Adult. *N* = 107 pts divided for symptoms in severe, critical, moderate, mild, asymptomatic, hospedalized Non-COVID-19.	*N* = 15 severe *N* = 17 critical pts were intubated	*N* = 32 Moderate; *N* = 26 mild; *N* = 12 asymptomatic	*N* = 5 subjects	ND	*N* = 24 subjects; *N* = 12 Healthy volunteers administered with intravenous lipopolysaccharide (IV-LPS) as a surrogate for an acute systemic inflammatory response
	males = females	Severe MA: 54 y; Critical MA: 54 y	Moderate MA: 54 y; Mild MA: 53 y. Asymptomatic MA: 50.5 y	MA: 56 y		MA 55.5 y
	MND: for severe 15 d; for critical 12.7 d; for moderate 10.5 d; for mild 10 d.	3/4 samples were females in critical group. 5/7 samples were female for severe group	11/17 males in moderate group.6/11 females in mild group			6/12 males in IV-LPS
**Vigon 2021 Frontiers in Immunology [45]**	Adult. *N* = 109 pts divided in 3 groups based on the symptoms.	*N* = 19 Severe *N* = 35 Critical (27/35 IMV); 13% of severe and critical pts developed DIC.	*N* = 55 pts with mild symptoms and who did not develop DIC	ND	ND	*N* = 20 subjects with similar age and gender distribution as the pts with COVID-19
	↑males↓females	MA: 72 y for Severe. MA: 63 y for Critical.	MA: 46 y			MA: 55.5 y
	MDH: 23 d severe pts; MDH: 50 d critical pts.	63% males for Severe pts and 74% for Critical	67% females			32.7% females
**Zhang 2020 Nature Immunology [46]**	Adult. *N* = 13 pts classified into moderate, severe and convalescent.	*N* = 4 pts in ICU	*N* = 7 pts	ND	*N* = 6 pts but only 4 were paired with moderate pts	*N* = 5
	↑ males↓females	MA: 64 years	MA: 37 y		MA: 42 y	MA: 35 y
	Mean of days after onset of symptoms: 6 d	50% males	57% males		66% males	100% males
**Chen 2021 Signal Transduction and Targeted Therapy [47]**	Women. *N* = 179 pts composed by pregnant and non-pregnant COVID-19 pts.	*N* = 20 PCov; 60% IMV. 5% Non-IMV *N* = 4 enrolled in the single cell study and divided in two subgroups: PCovM and PCovS	Only one was asymptomatic and her age was higher than others (43 y)	ND	*N* = 4 Pcov pts	*N* = 4 PHC pregnant healthy controls
		MA: 30.5 y			.	MA of PHC: 32 y
	Hospital staying: 16.5 d for pregnant and 14 d for Non-pregnant pts.	*N* = 23 NPCov 14% of NP showed critical symptoms; *N* = 6 enrolled in the single cell study	*N* = 136 NPCov		*N* = 6 Ncov pts	*N* = 6 NHC. Only *N* = 3 enrolled in the single cell study
		MA: 33 y				MA of NHC: 36 y

AD: Acute disease; COVID-19: Coronavirus Disease-19; DIC: Disseminated intravascular coagulation; ICU: Intensive care Unit; IMV: Invasive mechanical ventilation; LPS: Lipopolysaccharides; MA: Mean age; MDH: Median days of hospitalization; MDS: Median duration of symptoms before admission in ICU; MND: Median number of days from clinical onset to sampling; *N:* Number; NHC: Nonpregnant healthy controls; NP: Nonpregnant patients; NPCov: Nonpregnant COVID-19 patients; NPCovM: Nonpregnant COVID-19 patients with Moderate symptoms. NPCovS: Nonpregnant COVID-19 patients with Severe symptoms. PCovM: Pregnant COVID-19 patients with Moderate symptoms; PCovS: Pregnant COVID-19 patients with Severe symptoms; pts: Patients; y: Years.

**Table 2:** Characteristics of cohort of COVID-19 patients in studies on MAIT cells.

	Adult/Child	Severe	Non-Severe	Non-COVID-19 in ICU	Recovered	Healthy Subject
**Chen 2021 Signal Transduction and Targeted Therapy [47]**	Women. *N* = 179 pts composed by pregnant and NPCov. Hospital staying: 16.5 d for pregnant and 14 d for NP pts.	*N* = 20 pts. 60% IMV. 5% Non-IMV. *N* = 4 enrolled in the single cell study as PCovM and PcovS.	Only one was asymtomatic and her age was higher than others (43 y) *N* = 136 NPCov	ND	*N* = 4 Pcov pts	*N* = 4 PHC MA of PHC: 32 y;
		MA 30.5 y				
		*N* = 23 NPCov 14% of NP showed critical symptoms;			*N* = 6 Ncov pts	*N* = 6 NHC. Only *N* = 3 enrolled in the single cell study MA of NHC: 36 y.
		MA: 33 y				
**Deschler 2021 Viruses [43]**	Adult. *N* = 43 hospitalized COVID-19 pts.	*N* = 21 pts in ICU; 19/21 pts (90%) IMV.	*N* = 22 pts	ND	Sampling: 4–9 weeks after admission to the hospital.	*N* = 25
	↑ males↓females	MA: 65 y	MA: 62 y			MA: 28 y
		76.2% males	54.5% males			52.0% males
**Hubrack 2021 Scientific report [48]**	Adult. *N* = 36 pts classified based on the symptoms. ↑ males↓females	*N* = 13 pts 92.3% had difficulty of breath.	*N* = 23 pts 21.7% asymptomatic.	ND	ND	*N* = 21
		MA: 41 years	MA: 38.4 y			MA:38 y
		92% males	91.3% males			95% males
**Notarbartolo 2021 Science [49]**	Adult. *N* = 17 pts. For only 4 pts there are paired data of infection and post-infection.	*N* = 11pts. All required supplementary oxygen support.	*N* = 6pts	ND		*N* = 4
	↑ males↓females	MA: 55 y	MA: 33 y		Sampling: weeks after the resolution of infection.	
		54% females	83% males			
**Shi 2021 Frontier in Immunology [50]**	Adult. *N* = 13 pts classified in three clinical conditions. Samples data from the G. S. A. of the Beijing Institute of Genomics (BIG)	*N* = 4 pts MA: ND 50% males	*N* = 7 pts MA: ND ↑males	ND	*N* = 6 of whom 4 were paired with moderate cases.	*N* = 5
**Yu 2021 Med [51]**	Adult. *N* = 28 pts.	*N* = 9 pts. 77.8% requiring intensive care	*N* = 19 pts *N* = 11 pts paired with post-infection sampling.	*N* = 7 exposed subjects with β-phenotype.	. Sampling: weeks after the resolution of infection.	*N* = 10 Males with β-phenotype, females with α-phenotype.
	Mean of days after onset to hospitalization::	56% males	58% males	57% males		50% males
	8.5 d;	MA: 59,4 y	MA 36,7 y	MA: 42 y for exposed		MA: 39.7 y
**Yang The Journal of Immunology 2021 [52]**	Adult. *N* = 45 pts among mild and severe; *N* = 6 asymptomatic.	*N* = 22 pts. 23% required IMV	*N* = 23 pts MA: 41.7 y. More females than males.	*N* = 6 asymptomatic MA: 33 y.	*N* = 6 convalescent severe; N=8 convalescent mild pts.	*N* = 44
		54,5% males	43,7% males			
		MA: 55.7 y	MA: 41.7 y			MA: 33 y
**Flament 2021 Nature Immunology [53]**	Adult. **I cohort**: *N* = 182 pts in 3 groups: IDU(moderated) ICU (Severe) and deceased(Death)	*N* = 51 pts in ICU. 41% Death rate. MA:58 y 82.3% males	*N* = 51 pts. 11,7% death rate. MA: 61y 66,6% males	ND	ND	*N* = 80 MA: 27.2 60% males. *N* = 4 Uninfected MA: 58 y 75% males
	Adult. **II cohort**: *N* = 26 pts in 3 groups: IDU (moderated) ICU (Severe) and deceased (Death). ↑ males↓females for both cohorts	*N* = 13 ICU. 23% Death MA: 57 y 76,9% males	*N* = 9 IDU. 11% Death. MA: 79 y 55.5% males			
**Parrot 2020 Science Immunology [40]**	Adult. *N* = 69 pts with acute disease or convalescents from Atlas cohort or Biobank.	*N* = 15 samples from Atlas blood pts + *N* = 14 samples from Biobank.	*N* = 9 samples from Atlas blood pts	ND	*N* = 45 pts. *N* = 23 C. pts from mild disease. *N* = 22 convalescent pts from moderate/severe.	*N* = 14 Mild convalescent = 23; MA=51 y; 52% females. Moderate/severe convalescent= 22. MA= 56 y; 82% males.
	↑males↓females	MA: 57 y for both cohorts	MA: 56 y		Sampling within 1 to 6 weeks from resolution of disease.	
	Days in hospital: 17 d for Atlas and 34 d for Biobank;	80% males for Atlas samples	67% males.		48% males in mild convalescent; 82% males in moderate/severe convalescent.	
**Zhang 2021 Nature Immunology [46]**	Adult. *N* = 13 pts classified into: moderate, severe and convalescent.	*N* = 4 pts in ICU	*N* = 7 pts;	ND	*N* = 6 pts but only 4 paired with moderate pts;	*N* = 5 HCs;
	↑ males↓females	MA: 6 y	MA: 37 y		MA: 42 y;	MA: 35 y
	Mean of days after onset of symptoms: 6 d	50% males;	57% males;		66% males;	100% males
**Jouan 2020 JEM [39]**	Adult. *N* = 30 pts in ICU for severe COVID-19;	*N* = 30 pts in ICU; 66.7% pts IMV;	ND	*N* = 17 critically ill pts without pneumonia, requiring IMV.	*N* = 14 discharged pts to ICU ward.	*N* = 20 age and sex-matched
	↑ males↓females	MA: 64 y;		MA: 64 y;		
	Median duration of symptoms before admission in ICU: 10 d	75% males		55% males		

AD: Acute disease; COVID-19: Coronavirus Disease-19; DIC: Disseminated intravascular coagulation; ICU: Intensive care Unit; IMV: Invasive mechanical ventilation; LPS: Lipopolysaccharides; MA: Mean age; MDH: Median days of hospitalization; MDS: Median duration of symptoms before admission in ICU; MND: Median number of days from clinical onset to sampling; N: Number; NHC: Nonpregnant healthy controls; NP: Nonpregnant patients; NPCov: Nonpregnant COVID-19 patients; NPCovM: Nonpregnant COVID-19 patients with Moderate symptoms. NPCovS: Nonpregnant COVID-19 patients with Severe symptoms. PCovM: Pregnant COVID-19 patients with Moderate symptoms; PCovS: Pregnant COVID-19 patients with Severe symptoms; pts: Patients; y: Years.

**Table 3:** Characteristics of cohort of COVID-19 patients in studies on γδ cells.

	Adult/Child	Severe	Non-Severe	Non-COVID-19 in ICU	Recovered	Healthy Subject
**Odak 2020 eBioMedicine [41]**	Adult. *N* = 30 hospitalized COVID-19 pts	*N* = 15 pts with non-IMV;	*N* = 15 pts with stable parameters with no oxygen flow;	ND	*N* = 7 pts Sampling: weeks after the resolution of infection.	*N* = 60 Matched HCs
	↑ males↓females	MA: 60 y	MA: 68 y			Mean age: 54 y
	Mean of days after onset of symptoms: 11 d	86% males	73% males			80% males
**Zhang 2020 Nature Immunology [46]**	Adult. *N* = 13 pts: moderate, severe and convalescent.	*N* = 4 pts in ICU	*N* = 7 pts	ND	*N* = 6 pts but only 4 were paired with moderate pts;	*N* = 5 HCs;
	↑ males↓females	MA: 64 y	MA: 37 y		MA: 42 y	MA: 35 y
	Mean of days after onset of symptoms: 6 d	50% males	57% males		66% males	100% males
**Jouan 2020 JEM [39]**	Adult. *N* = 30 pts in ICU for severe COVID-19; ↑ males↓females Median duration symptoms before admission in ICU: 10 d	*N* = 30 pts in ICU 66.7% pts IMV MA: 64 y 75% males	ND	*N* = 17 critically ill pts without pneumonia requiring IMV Mean age: 64 years; 55% males	*N* = 14 discharged pts to ward. Sampling: 15 d	*N* = 20 subjects age and sex-matched
**Stephenson 2021 Nature medicine [44]**	Adult. *N* = 107pts: severe, critical, moderate, mild, asymptomatic, hospitalized Non-COVID-19.	*N* = 15 severe and *N* = 17 critical pts with IMV	*N* = 32 moderate; N=26 mild; *N* = 12 asymptomatic.	*N* =5 subjects MA: 5 y	ND	*N* = 24 *N* = 12 Healthy volunteers treated with intravenous lipopolysaccharide (IV-LPS)
	Males = females	Severe MA: 54 y; Critical MA: 54 y.	Moderate MA: 54 y; Mild MA: 53 y. Asymptomatic MA: 50.5 y.			MA: 55.5 y
	Mean of days from onset of symptoms: for severe 15d; for critical 12.7 d; for moderate 10.5d; for mild 10 d.	3/4 pts females in critical group. 5/7 pts female for severe group.	11/17 pts males in moderate group. 6/11 pts female for mild group.			6/12 pts males in IV-LPS

AD: Acute disease; COVID-19: Coronavirus Disease-19; DIC: Disseminated intravascular coagulation; ICU: Intensive care Unit; IMV: Invasive mechanical ventilation; LPS: Lipopolysaccharides; MA: Mean age; MDH: Median days of hospitalization; MDS: Median duration of symptoms before admission in ICU; MND: Median number of days from clinical onset to sampling; N: Number; NHC: Nonpregnant healthy controls; NP: Nonpregnant patients; NPCov: Nonpregnant COVID-19 patients; NPCovM: Nonpregnant COVID-19 patients with Moderate symptoms. NPCovS: Nonpregnant COVID-19 patients with Severe symptoms. PCovM: Pregnant COVID-19 patients with Moderate symptoms; PCovS: Pregnant COVID-19 patients with Severe symptoms; pts: Patients; y: Years.

**Table 4:** : Characteristics of cohort of COVID-19 patients in studies on ILCs cells.

	Adult/Child	Severe	Non-Severe	Non-COVID-19 in ICU	Recovered	Healthy Subject
**Garcia 2021 Clinical and translational Immunology [55] **	Adult. *N* = 23 pts; *N* = 16 HCs.	*N* = 12 pts in ICU with IMV	*N* = 11 pts; Non-intubated and Non-oxygen need. Some hospedalized ICU.	ND	ND	*N* = 16
	↑males↓females	MA: 59.5 y	MA: 56 y			
	Median days hospitalized: 11 d for moderate pts; 22 d for severe pts.	83% males	63.6% males.			
**Segundo 2020 Biomedicines [58]**	Adult. *N* = 150 pts divided based on oxygen therapy requirements.	*N* = 82 mod/sev (ICU with IMV or only hospitalized or deceased)	*N* = 73 Mild pts	ND	ND	ND
		MA: 72 y	MA: 59 y			
		↑ male gender.	↑ female gender.			
**Silverstein 2021 (preprint) [56]**	Adult cohort of samples	*N* = 40 pts. Among whom N=33 (82.5%) at ICU; N=32 (80%) with IMV; N=7 (17.5%) died.	*N* = 51 outpatients infected with SARS-CoV-2 who were treated for COVID-19.	ND	ND	*N* = 86 who donated blood prior to the SARS-CoV-2 outbreak.
	↓males ↑ females	MA: 57.6 y	MA: 36.8 y			MA: 50.9 y
	Mean days hospitalized: 34.2 d	60% males gender	25.5% males gender			55.8% males
	Pediatric. *N* = 30 pts; *N* = 17 HCs.	*N* = 11 pts. Among whom *N* = 1 (5.3%) at ICU with IMV	*N* = 8 outpatients infected with SARS-CoV-2	*N* = 11 MIS-C pts	*N* = 14 COVID-19 follow-up; *N* = 7 MIS-C follow-up; *N* = 10 pts (5 COVID-19 and 5 MIS-C)	*N* = 17 SARS-CoV-2-uninfected pediatric blood donors
	↑males ↓ females	MA: 13 y	MA: 13 y			
**Gomez-Cadena 2021 Cellular & Molecular Immunology [57]**	Adult. *N* = 60 pts hospitalized for COVID-19. males = females	*N* = 30 pts MA: 68.3 with 41% of the severe pts were older than 75 y	*N* = 30 with mild symptoms. MA: 39.8 y	ND	ND	*N* = 21
	Pts were enrolled at least 21 d after the first symptoms of COVID-19	76.7% males	23.3% males			
**Gomez 2021 Eur. J. Immunology [58]**	Adult. *N* = 20 pts hospitalized for COVID-19. Days of symptoms to admission: 12 d.	*N* = 20 pts 11/20 pts in ICU 80% males MA: 56 y	ND	ND	*N* = 14 pts in follow-up. Days of symptoms to recovery: 19 d	*N* = 9MA: 58 y

AD: Acute disease; COVID-19: Coronavirus Disease-19; DIC: Disseminated intravascular coagulation; HC: healthy control; ICU: Intensive care Unit; MDS: Median duration of symptoms before admission in ICU; IMV: Invasive mechanical ventilation; LPS: Lipopolysaccharides; MA: Mean age; MDH: Median days of hospitalization; MIS-C: Multisystem Inflammatory Syndrome in Children. MND: Median number of days from clinical onset to sampling; N: Number; NHC: Nonpregnant healthy controls; NP: Nonpregnant patients; NPCov: Nonpregnant COVID-19 patients; NPCovM: Nonpregnant COVID-19 patients with Moderate symptoms. NPCovS: Nonpregnant COVID-19 patients with Severe symptoms. PCovM: Pregnant COVID-19 patients with Moderate symptoms; PCovS: Pregnant COVID-19 patients with Severe symptoms; pts: Patients; SARS-CoV-2: Severe acute respiratory syndrome coronavirus 2; y: Years.

## Supplementary Materials

The following are available online at https://www.mdpi.com/article/10.3390/cells11030542/s1, Table S1: Phenotypic and function alterations of NKT cells in SARS-CoV-2 infection; Table S2: Phenotypic and function alterations of γδ T cells in SARS-CoV-2 infection; Table S3: Phenotypic and function alterations of ILCs in SARS-CoV-2 infection; Table S4: Phenotypic and function alterations of MAIT cells in SARS-CoV-2 infection.
