# Supplementary material for: Comprehensive Analysis of the ILCs and Unconventional T Cells in Virus Infection: Profiling and Dynamics Associated with COVID-19 Disease for a Future Monitoring System and Therapeutic Opportunities
Source: Cells. 2022 Feb 4;11(3):542. doi: 10.3390/cells11030542 (PMC8834012; doi:10.3390/cells11030542)
Supplement: Supplementary file 1 [file cells-11-00542-s001.zip › cells-1514208-supplementary.pdf]

Table S1: Phenotypic and function alterations of NKT cells in SARS-CoV-2 infection.

	Type of sample	Activated mediators	Produced Cytokines	In situ recruitment	Chemokine receptors	Expression Markers	Differential expression markers in subsets	
**Jouan et al (Ref 39)**	Serum, Blood, ETAs. Tool: Flow cytometry.	↑ level of IL-1β, IL-6, IL-1RA, IFNα2, IFNγ, IL17 in supernatants than serum.	Upon PMA/ Iono: ↑ % IL17 NKT cells than IFNγ NKT in COVID-19 pts compared to HCs.	Airway NKT cells undetectable	In ETAs: ↑CXCL10, CXCL12	PD-1; CD69	Plasmatic IL18 Corr.+ with CD69 NKT in COVID-19 pts.	↑ PD1 NKT in COVID-19 pts compared to HCs.
		↑IL18 in COVID-19 pts.					↑CD69 in COVID-19 pts compared to HCs and Non-COVID-19.	
							CD69 NKT Corr.+ with decreasing hypoxemia.	
**Parrot et al (Ref 40)**	Serum, Blood. Tool: Flow cytometry; Sc-RNA-seq.	↑CXCL10, CX3CL1 in serum of COVID-19 pts compared to HDs.	ND	ND	ND	ND	ND	ND
**Odak et al (Ref 41)**	Blood analyzed by Flow cytometry	ND	ND	ND	ND	ND	ND	ND
**Tomi et al (Ref 42)**	Blood, Serum; Tool: Flow cytometry	In severe: ↑ IL-6, IL-8, MCP-1, IL-18, TGFβ, IL-10. ↓IL-12p70.	ND	ND	ND	ND	ND	ND
		In mild: ↓IL-10 , IL4. ↑TGFβ. ↓ IL-17A, IL-17F.						
**Vigon et al (Ref 45)**	Blood, Serum; Tool: Flow	Plasma of Critical: ↑IL-8/ CXCL8, IL-6, TNFα; IFNγ;	ND	↓%CD56+CD16+GZB+ in response to Hsp70 peptide.	ND	Degranulation and	↑% CD3+CD56+CD16+ in C.C.	↑% CD3+CD56+CD16+ CD107a+ in C.C. in

	Cytometry, Luminex.	Plasma Severe and Critical: ↑CD25/IL-2Ra; ↓ IL2, compared to mild disease.		↓cytotoxic capacity against target cells without MHC class I molecules in comparison with M.C.		cytotoxic markers.		comparison with mild (data not significant).

Surnatant of endotracheal aspirates: *ETAs;* Positively correlated: *Corr.+;* critical COVID-19: *C.C.;* mild COVID-19: *M.C;* ↑ increases frequency or number compared to Controls; ↓ decreases frequency or number compared to Controls. Patients: *pts*.

Table S2: Phenotypic and function alterations of γδT cells in SARS-CoV-2 infection.

	**Type of sample**	**Activation marker in blood**	**Activation marker in situ**	**Expression marker**	**Differential expression markers in subsets**			
**Odak et al (Ref 41)**	Blood; Tool: Flow cytometry	ND	↓% γδ eff cells in blood	CD45RA, CD62L	↑CD45RA+CD62+ (γδ naïve).	↓CD45RA-CD62- (γδ eff).	↓CD45RA-CD62L+ memory- in Severe patients compared to HCs.	↓CD45RA+CD62L- in Severe patients compared to HCs.
**Zhang et al (Ref 46)**	Blood; Tool: Sc-RNA-seq	ND	ND	Genes: GZMK GZMA PRF1 TRAV1-2 TYROBP	ND			
**Jouan et al (Ref 39)**	Serum, blood and surnatant of ETAs. Tool: Flow cytometry.	↑%CD69+ γδ T cells, % PD1+ γδ T cells compared to HDs.	↑%CD69+ γδ T cells, PD-1+ γδ T cells in ETA compared to blood of matched pts	ND	Vδ2+: ↑ CD69+ compared to HDs. ↑ PD1 compared to HDs.	CD69+ Vδ1: from COVID-19 pts were not significantly changed compared with non–COVID-19 ill controls. ↑ PD1+ Vδ1 compared to HDs.	Vγ1Vδ2-: from COVID-19 pts were not significantly changed compared with non–COVID-19 ill controls. PD1+ Vγ1Vδ2-: did not change compared to HDs.	

**Stephenson et al (Ref 44)**	Blood, Serum; Tool: gene expression.	IL1B, IL1A and TNFα.	ND	↓ CD28, CTLA4, CD40GL	ND

Effector-like γδ cells; ↑ increases frequency or number compared to Controls; ↓ decreases frequency or number compared to Controls.

Table S3: Phenotypic and function alterations of ILCs in SARS-CoV-2 infection.

	**Type of sample**	**Activation marker in blood**		**Differential expression markers in subsets**	
**Garcia et al (Ref 55)**	Blood analyzed by Flow cytometry	Levels of the cytokine IL- 10 positively correlated with the levels of CD69+ ILCs in the COVID-19 patients.	ND	↓ CXCR3. CCR4–/lo ILC1 clusters were overrepresented in severe COVID-19 patients.	↓CD56+ ILC1 in COVID-19 patients.
				ILC2 negatively correlated with CCL20 levels.	↑CD69+ ↓ CD62L+
		Activated ILCp positively correlated with serum IL-6 levels in the COVID-19 patients.		ILCp positively correlated with CXCL10 levels in the COVID-19 patients. ↓CXCR3+ activated ILCp in both moderate and severe COVID-19.	↑CD69+ ; (CD45RA+/hi/CD62L+/hi) were reduced in COVID-19 pts as compared with controls. IL18R1 and PD-L1 negatively correlated with the level of CD45RA+ ILCp.
**Segundo et al (Ref 58)**	Blood, serum; Tool: Flow cytometry.	IL6	ND	ND	ND
**Silverstein et al (Ref 56)**	Blood analyzed by Flow cytometry.	ND	ND	ND	ND
	RNA-seq profiles of ILC from available dataset online	ND	ND	ND	ND
	Blood analyzed by Flow cytometry.	Males had a lower median fraction of AREG+ ILCs than females. AREG expression in males was lower than females.	IL13 and amphiregulin (upon in vitro stimulation with Iono/PMA).	They hypothesis that ILC2 were recruited in lung tissue. They compared RNA-seq on blood ILCs from 9 HCs to previously	ND

				published RNA-seq profiles of ILCs sorted from lung, spleen and Intestine.	
	RNA-seq profiles of ILC from available dataset online	ND	ND	ND	ND
**Gomez-Cadena et al (Ref 57)**	Mouse model and Blood from patients; serum analysis.	Elevated levels of the type 2 cytokines IL-5 , IL-13 and significant increase in IL-33 in COVID-19 patients compared to that in healthy donors; in particular serum IL-18 and IL6 levels were significantly higher in severe COVID-19.	ND	ND	ILC2s in severe pts ↑ NKG2D+ population compared to those in mild pts and controls, CD25 and KLRG1 ↓. No differences in NKG2D, KLRG1, or CD25 expression were observed in ILC1s or ILCPs. PD-1, NKG2A, and NKp46, were similar on ILC2s from HDs and pts.
	ND	ND	ND	ND	ILC2 NKG2D expression: Low 31.2%, High 68.18% of severe pts.

↑ increases frequency or number compared to Controls; ↓ decreases frequency or number compared to Controls. Patients: *pts*.

Table S4: Phenotypic and function alterations of MAIT cells in SARS-CoV-2 infection.

	Type of sample	Activated mediators	Produced Cytokines	In situ recruitment	Chemokine receptors	Expression Markers	Differential expression markers in subsets	
**Chen et al (Ref 47)**	Blood; Tool: sc-RNA seq and TCR sequencing	↑*IFNGR1, STAT1, TNFRSF1B, TNFAIP3,* and *TNFSF8* in Pcov (Severe, moderate and healthy) compared to Non-Pregnant (Severe, moderate and healthy) significantly.	ND	GO terms of “leukocyte cell–cell adhesion” was pronouncedly enriched in PCov groups than in the NCov groups compared to their corresponding HCs.	↑CXCR4 in PCov groups compared to NHC.	ND	ND	ND

	Type of sample	Activated mediators	Produced Cytokines	Expression Markers
**Deschler et al (Ref 43)**	Blood; Tool: Flow Cytometry	↓ IL-12R and IL-18R from COVID-19 patients compared to healthy controls.	Upon in vitro E.coli stimulation, significantly ↑ IFNγ expression; MAIT cells failed to upregulate expression of IL-17A and TNFα. Upon in vitro stimulation with IL-12 and IL-18, ↓IL-17A and TNFα from COVID-19 pts compared to healthy controls; ↑ IFNγ in response to IL-12/IL-18 ↓ in response to E. coli stimulation. Any differences in the expression of TNFα and IL-17A among mild and severe COVID-19 irrespective of the way of stimulation.	Expression of CD38, CD69 and HLA-DR, CTLA-4 and PD-1 in COVID-19 patients compared to healthy controls. Precisely, ↑CD38 and CD69 significantly in acute COVID-19 than Convalescent.<br>ND
			↓ granzyme B expression and perforin expression upon IL-12/IL-18 stimulation. MAIT from severe COVID-19 were able to significantly ↑ granzyme B expression upon IL-12/IL-18 stimulation and significantly higher levels from patients with mild COVID-19.	↑CTLA-4 and PD- 1 in severe COVID-19 but not significantly. ↓CTLA-4 in convalescent patients with severe COVID-19 and ↓↓ CTLA-4 in mild COVID-19.
			Ex vivo analysis: ↑ TNFα and IL-17A, GRZ B in Covid-19 compared to healthy controls while IFNγ and perforin were unchanged. ↑Perforin in patients with severe COVID-19 compared to mild COVID-19 and healthy controls; IFNγ, TNFα, IL-17A and GRZ B did not differ between MAIT cells isolated from patients with mild or severe COVID-19.	
**Hubrack et al (Ref 48)**	Blood; Tool: Flow Cytometry.	ND	Upon in vitro long stimulation with IL12/IL18: ↓ GRZ B, TNF, IFNγ, Perforin compared to controls.	↑CD69 (% and MFI) in Severe patients compared to HC.
			Upon in vitro stimulation with IL7: ↓ GRZ B, IFNγ, Perforine but not significantly. ↓ TNFα significantly.	Slight decrease level of CD69 (% and MFI) in mild patients compared to severe. Data did not significant.
			Upon in vitro IL12/IL18 or IL7: ↑ MFI of Perforin compared to Unstimulated condition.	
**Notarbartolo et al (Ref 49)**	Blood. Tool: Sc-RNAseq and TCR-seq;	↑CD69, FOS, DUSP1	↑ IFN- responsive genes in patients with severe disease.	*KLRB1 (CD161), SLC4A10, RORC, and CCR6* *TCR* gene usage *(TRAV1-2, TRAJ33/12/20, and TRBV20/6)*<br>*CCL5, NKG7, PRF1, CST7, and GZMK*

	Flow cytometry.					
	Type of sample	Chemokines	**Expression markers**	**Differential expression markers in subsets**		
**Shi et al (Ref 50)**	scRNA-seq raw data from GSA	↑ genes associated with chemotaxis and apoptosis in severe cases.	↑*TRBV9, TRAV8-2, S100A8, GZMH, S100A9, KLF6, CD8B, KLRD1, IGLV3-19,* and *JCHAIN*<br>↓ *SLC4A10* and *TRAV1-2*<br>The number of high frequencies of clonotypes of MAIT cells was relatively high. There were more large clonal expansions (clonal size >10) in the severe cases than in the other conditions. ↓The expression levels of genes associated with cell activation in MAIT of severe cases.	↑% TRAV1–2 or TRBV33 cells. ↓ TRAV1-2 or TRBV33 compared with moderate and convalescent cases.	↓TRAV1-2 in severe cases compared with other conditions.	↓The usage level of gene pair TRAV1-2 and TRAJ12 in severe cases.

	**Type of sample**	**Cytokine production by Flow Cytometry analysis**	**Cytokine production by Sc-gene expression**			
**Flament et al (Ref 53)**	Blood analysed by flow cytometry. Serum to quantify proteins.	In the blood of surviving patients with COVID-19 hospitalized in an IDU: ↓IL-6, IL-8, IL-10, ↑IFNα2, ↓IL15, IL18, IL1β compared to ICU o fatal Covid-19.	Upon Iono/ (PMA) ionomycin in vitro stimulation and withou stimulation:↑ IFNγ, IL2 and GZB and IL17 of circulating MAIT in severe patients.			
		In the blood of surviving patients with COVID-19 hospitalized in an ICU: ↓IL-6, IL-8, IL-10, IFNα2, ↓IL15, IL18, IL1β compared to fatal Covid-19.	↑IFNγ and TNFα and GzB in deceased patients compared to surviving patients.			
	Blood analysed by Facs Sorting and RT-qPCR. Sc-gene expression.	In the blood of surviving patients with COVID-19 hospitalized in fatal COVID-19: ↑IL-6, IL-8, IL-10, IFNα2, IL15, IL18, IL1beta compared to ICU and IDU except for IFNγ. Note: differences in IL1b are not significant.	Gene expression in ICU: ↓ *IFNA* ↑ *IL18* in opposite compared to IDU. Gene expression in ICU: ↓ *STATA1, IRF1, IRF9* compared to IDU. Gene Expression in ICU: ↑*NLRP3, TXNIP* compared to IDU. Gene Expression in ICU: ↑*PRDM1, HOBIT, TBX21.*			
**Yang et al (Ref 52)**	Blood analyzed by Mass cytometry and Flow Cytometry.	Upon E. coli stimulation or IL12/IL18: severe COVID-19 ↓IFNγ, GzmB, and CD107a than those from HC; Upon coculture with CD14+ cells from patients with severe COVID-19 ↓IFNγ and GzmB production.	TEM (CD45RA + CD197-).	naive (CD45RA + CD1971+)	central memory T (CD45RA	TEM (CD45RA- CD197 +) like cells.

		Upon E.coli stimulation or IL12/IL18: mild COVID-19 ↓ GzmB and CD107a than those from HC.		. Significantly decreased in patients with severe COVID-19	- CD1971-). Increase in both Mild and Severe COVID-19 patients.	Significantly increased in patients with mild and severe COVID-19.
		In asymptomatic carriers with COVID-19 function of MAIT remain unchanged. Upon E.coli stimulation or IL12/IL18: convalescent patients restored their functions.				

	Type of sample	In situ recruitment	Expression markers	Differential expression markers in subsets	
**Yu et al (Ref 51)**	Blood, BALF, NPS. Tool: Flow cytometry; sc-RNA seq.	↑MAIT in airway tissue of Covid-19 Females more than males.	IL-4, IL-13, IL-10, TGF-b, NGF- TRKA signaling axis, RAF-independent mitogen-activated protein kinase (MAPK), estrogen-dependent pathway.	MAIT alpha in healthy individuals but after SARS-CoV2 infection females pts lost this phenotype.	MAIT beta in exposed and infected groups; females in late disease increased percentage of phenotype beta.
		airway tissue of Covid-19 Females: *IL7R, CISH, SOCS1.* ↑ *BCL2, FOXP1, CDKN1B,BTG2, KLF2, MYC*, and *CEBPD.* ↓*BAX* and *CASP3*	↑CD69 in hospitalized group without any correlation with sex or phenotype.		
		airway tissue of COVID-19 Males: ↑CCL2.			
**Parrot et al (Ref 40)**	Serum, blood. Tool: Flow cytometry and Sc-RNA-seq.	The transcriptional profile indicated that MAIT cells were the main subset of airway T cells expressing IL17A. This profile was paired with expression of TNF and an apparent lack of IFNG and GZMB transcripts.	Inverse correlation between CCL28, CXCL11, CCL20 , IL17C and absolute number of MAIT.	↑CD69 in AS patients together with GrzB and Ki67 than AM patients . PD-1, IL-7R, CXCR6, granzyme A (GrzA), and CD56 were similar between HD, AM, and AS groups.↑ CD69 on MAIT cells of died patients (analyzed 4 patients) than patients	

				who survived. In convalescent patients CD69 repristinated its normal values.	
	ND	↓CXCR3 in both AM and AS COVID-19 patients. In convalescent patients, CXCR3 levels were still suppressed.	ND	ND	ND
**Zhang et al (Ref 46)**	Blood; Tool: Sc-RNA-seq	ND	ND	GO analysis demonstrated that MAIT from moderate and severe patients responded more to IFN than the acute inflammation.	
	**Type of sample**	**Cytokine expression in situ**	**Cytokine expression in blood**	**Differential expression markers**	
**Jouan et al (Ref 39)**	Serum, blood and surnatant of ETAs. Tool: Flow cytometry.	↑% MAIT in the airways.↑ IL-1β, IL-6, IL-1RA, IFN-α2, IFNg and IL17 in supernatants of ETAs. The plasma level of IL18 ↑ in Covid-19 pts.	Circulating MAIT produced less IFNγ compared to HCs and increase capacity to produce IL17A.	CD69+ MAIT positively correlated with the level of plasmatic IL18. ↑CD69, PD1 MAIT in ETA compared to blood of the same patients.	

Non-Pregnant healthy controls: *NHC*; Genome Sequence Archive: *GSA;* Bronchoalveolar lavage fluid: *BALF*; nasopharyngeal swabs: *NPSs*;
